# The ménage à trois of healthcare: the actors in after-AI era under patient consent

**DOI:** 10.3389/fmed.2023.1329087

**Published:** 2024-01-08

**Authors:** Riccardo Saccà, Rachele Turrini, Francesco Ausania, Stefania Turrina, Domenico De Leo

**Affiliations:** Department of Diagnostics and Public Health, Section of Forensic Medicine, University of Verona, Verona, Italy

**Keywords:** artificial intelligence, informed consent, therapeutic alliance, patient-caregiver relationship, medical ethics, patient autonomy

## Abstract

**Introduction:**

Artificial intelligence has become an increasingly powerful technological instrument in recent years, revolutionizing many sectors, including public health. Its use in this field will inevitably change clinical practice, the patient-caregiver relationship and the concept of the diagnosis and treatment pathway, affecting the balance between the patient’s right to self-determination and health, and thus leading to an evolution of the concept of informed consent. The aim was to characterize the guidelines for the use of artificial intelligence, its areas of application and the relevant legislation, to propose guiding principles for the design of optimal informed consent for its use.

**Materials and methods:**

A classic review by keywords on the main search engines was conducted. An analysis of the guidelines and regulations issued by scientific authorities and legal bodies on the use of artificial intelligence in public health was carried out.

**Results:**

The current areas of application of this technology were highlighted, divided into sectors, its impact on them, as well as a summary of current guidelines and legislation.

**Discussion:**

The ethical implications of artificial intelligence in the health care system were assessed, particularly regarding the therapeutic alliance between doctor and patient, and the balance between the right to self-determination and health. Finally, given the evolution of informed consent in relation to the use of this new technology, seven guiding principles were proposed to guarantee the right to the most informed consent or dissent.

## Introduction

The authors of this article believe it is necessary to pose an initial axiomatic consideration from which the subsequent reasoning can then be developed: “artificial intelligence is already a current reality, destined to become an integral part of the care process for doctor and patient, so it cannot be scotomized.”

Due to the multiple fields of application and underlying methods, it is not possible to date to give an unambiguous definition of artificial intelligence (A.I.) (1). In generic terms, A.I. is an iterative learning model based on the acquisition of big data that leads to the development of interpretations, predictive models and decision-making processes, not based on *a priori* mechanisms or dependent on third-party intervention (1). A specific definition of AI in a recommendation of the Council on Artificial Intelligence of the OECD states, “An AI system is a machine-based system that can, for a given set of human-defined objectives, make predictions, recommendations, or decisions influencing real or virtual environments. AI systems are designed to operate with varying levels of autonomy” (2). Machine learning is defined as the ability of the machine to learn without being programmed in advance (3). This process is the basis for the development of so-called A.I. based prediction models (AIPM), which are models that provide probabilistic predictions and outcomes after certain inputs have been provided (4).

As a testament to A.I. as an established reality and not a mere future possibility, suffice it to consider that ChatGPT, since its release in November 2022, with 100 million users in 2 months has represented an unprecedented spread in the technology world (5).

The areas of A.I. use, both in the public and private sectors, are many and rapidly increasing, to the point that, in part because of the scope and implications of this tool, the High-level expert group on artificial intelligence has been set up by the European Commission 2020 (1).

This unprecedented technological tool has already seen its reflection in multiple fields (e.g., finance, data processing, word processing and document analysis), including healthcare, laying the groundwork for a revolution in the system of care. Suffice it to say how several studies have tested AI’s diagnostic-interpretive capabilities in radiology, noting that these are equal to, if not superior to, those of experts in the field (6–9).

Moreover, the current era is experiencing a phase of innovation and implementation not only limited to the process of care, but also seeing the tools used in the same involved.

In fact, the technological revolution is also being reflected in the pharmaceutical and healthcare device sectors. In the former case, AI is already being applied in new drug discovery and development, clinical and nonclinical research processes, and post-marketing safety monitoring (10). In the second case, there is evidence that more and more devices using machine learning-based technology are being approved by the U.S. Food and Drug Administration (FDA) (11).

The relationship of care between physician and patient has undergone profound change over time, partly secondary to technological development and social and cultural changes over the centuries. From the earliest days of medicine, said relationship was characterized by a hierarchical setting, which saw the act of care and consequently the figure of the one who provided the care (priest, shaman and later doctor) as imposed on the patient, regardless of his or her will or opinion on the matter, in order to ensure the patient’s health or the good of the community. In this context, knowledge and choices regarding treatment were not accessible to the sick person, since, according to a paternalistic approach to the care relationship, he or she passively followed the course of care according to a pattern of vertical imposition (12). A pivotal example of this system is represented by Hippocratic medicine, where the explicit consent of the patient was not required, but the establishment, by implication, of a fiduciary relationship with the caregiver and in his or her presumed ability to be able to provide the necessary care was sufficient, believing that the principle of beneficence took priority over that of autonomy. After centuries, the doctor-patient relationship has evolved, transcending from the inherent subservience to care, with the end of paternalism and the emergence of the concept of “therapeutic alliance” (13), in which the value of autonomous decision-making on the part of the patient is affirmed, thus integrating the right to health with the right to self-determination.

In all the historical phases just described, at any rate, the relationship between patient and caregiver was a bipolar, uni- or bidirectional interaction between two human being or sentient entities. With the introduction of AI, however, this relationship is bound to change, with the addition of a third actor, the AI: this inevitably results in a paradigm shift, as the process of patient caretaking and the care pathway will be characterized by a triangulated interactive dynamic.

The advent of this third actor in the era of the therapeutic and post-paternalistic alliance implies inevitable repercussions on the foundational elements of the contemporary doctor-patient relationship: access to care and informed consent/dissent to care by the patient.

The concept of informed consent was introduced in the early twentieth century, when Judge Cardozo expressed himself regarding the patient’s right to self-determination, affirming the right for every adult with common-sense to dispose of his or her own body (14). In the years to come, the jurisprudential-ethical entity of informed consent further took root in the Nuremberg Code (1947), in which the principles underlying the lawfulness of health treatments and clinical trials are expressed, as well as further in the Declaration of Helsinki (1964), concerning medical research. This historical development reached synthesis with the drafting of the well-known Oviedo Convention (Convention of Human Rights No. 164), in which, Art. 5 states the following: “An intervention in the health field may only be carried out after the person concerned has given free and informed consent to it. This person shall beforehand be given appropriate information as to the purpose and nature of the intervention as well as on its consequences and risks. The person concerned may freely withdraw consent at any time” (15).

Already, therefore, the issue that appears necessary to be addressed is how and whether the patient will consent to AI co-participation in his or her course of care.

The aim of the classic review was to characterize the current role of AI in public health, as well as its future implications, by analyzing current areas of application, regulatory guidelines for use and current relevant legislation, to understand the actual interactions of AI with the doctor and the patient. This served as a basis for proposing key principles for informed patient consent to the use of AI.

## Materials and methods

A classic review of the scientific literature was conducted, using the main search engines such as Pubmed and Google Scholar. Keywords used included: “A.I.,” “informed consent,” “guidelines,” “machine learning,” “healthcare,” “medical devices,” “therapeutic alliance.” Subsequently, documents issued by national and international institutional control bodies as sources have been analyzed, in order to study the current guidelines and regulations in the field of the use of AI in public health, released by: FDA, World Health Organization (WHO), Health Canada, United Kingdom’s Medicines and Healthcare products Regulatory Agency (MHRA), American Medical Informatics Association (AMIA), International Coalition of Medicines Regulatory Authorities (ICMRA) and the European Parliament.

## Results

The classic literature review conducted on Pubmed and Google Scholar highlighted the areas of current application of AI, studies evaluating the efficacy and impact of its use, and the ethical implications of its presence in public health. The analysis of institutional sources (FDA, WHO, Health Canada, MHRA, AMIA, ICMRA and European Parliament) highlighted current guidelines on the design, use and monitoring of AI in public health, as well as current regulatory legislation.

Those are the results obtained from the review of the relevant scientific literature, as well as what emerged from the analysis of the main guidelines found and the relevant legislation.

**Figure 1:**
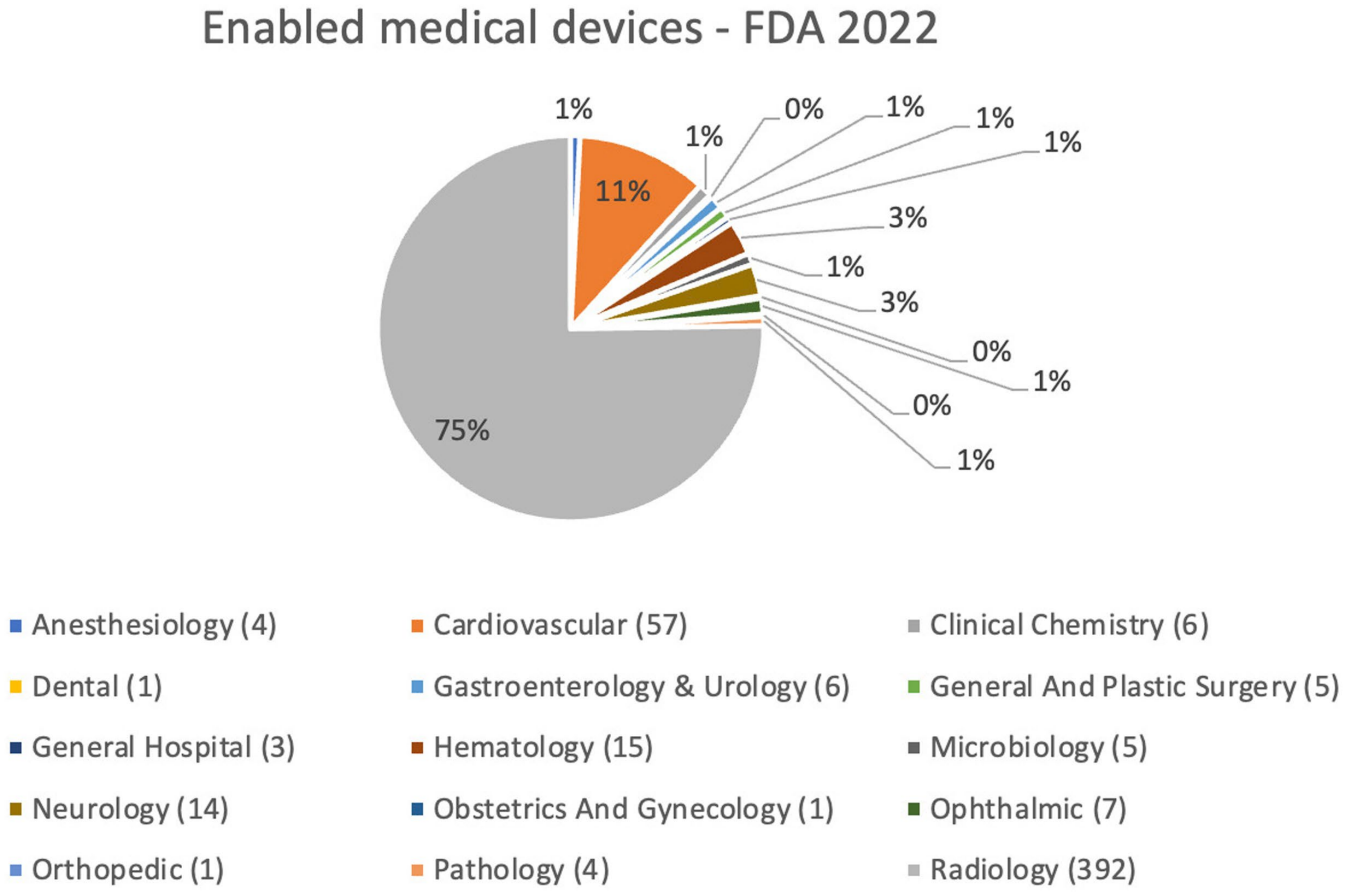
Enabled medical devices approved by FDA up to October 5, 2022.

As shown in Figure 1, by analyzing FDA databases, it was found that, to date, 521 biomedical devices have been approved in various application areas, including: 4 devices in anesthesiology; 1 dental; 3 general hospital; 14 neurology; 1 orthopedic; 57 cardiovascular; 6 in gastroenterology and urology; 15 hematology; 1 obstetrics and gynecology; 4 pathology; 6 clinical chemistry; 5 general and plastic surgery; 5 microbiology; 7 ophthalmology; and finally, a particular significance is observed in the approved devices in radiology, amounting to 392 (75% of total devices) (16).

From a study by de Hond et al. (4) existing guidelines and quality criteria regarding the development, evaluation and implementation phases of AIPMs were extrapolated and resumed in Table 1.

**Table 1:** Stages of AIPM development, evaluation and implementation.

Phase	Activity
Phase 1	Preparation, collection, and checking of the data
Phase 2	Development of the AIPM
Phase 3	Validation of the AIPM
Phase 4	Development of the software application
Phase 5	Impact assessment of the AIPM with software
Phase 6	Implementation and use in daily healthcare practice

They analyze best practices to be applied in the development of AIPMs in order to reduce the introduction of systematic bias, so as to optimize the yield and consequent benefits of applying these models in clinical practice.

Guiding principles proposed by the world’s leading institutional bodies for the development and use in healthcare of AI were also identified.

WHO compiled the first global report on AI in health (17), within which laws, policies and principles that apply to use of artificial intelligence for health are analyzed, as well as the ethical principles underlying its use, namely, “Protect autonomy,” “Promote human well-being, human safety and the public interest,” “Ensure transparency, explainability and intelligibility,” “Foster responsibility and accountability,” “Ensure inclusiveness and equity,” and “Promote artificial intelligence that is responsive and sustainable.”

The shared work operated by FDA, Health Canada, MHRA resulted in the identification of 10 guiding principles for the development of Good Machine Learning Practice (GMLP) (18), as shown in Table 2.

**Table 2:** Guiding principles for the development of GMLP.

Multi-Disciplinary Expertise Is Leveraged Throughout the Total Product Life Cycle.
Good Software Engineering and Security Practices Are Implemented
Clinical Study Participants and Data Sets Are Representative of the Intended Patient Population.
Training Data Sets Are Independent of Test Sets
Selected Reference Datasets Are Based Upon Best Available Methods.
Model Design Is Tailored to the Available Data and Reflects the Intended Use of the Device
Focus Is Placed on the Performance of the Human-AI Team
Testing Demonstrates Device Performance During Clinically Relevant Conditions
Users Are Provided Clear, Essential Information
Deployed Models Are Monitored for Performance and Re-training Risks Are Managed

Badal K. et al. (19), collected guidance from regulatory principles (Table 3) so far produced by the FDA, Health Canada (19), WHO (18), and AMIA (20).

**Table 3:** Regulatory principles produced by FDA, Health Canada, WHO, AMIA.

Al tools should aim to alleviate existing health disparities
Outcomes of Al tools should be clinically meaningful
Al tools should aim to reduce overdiagnosis and overtreatment
Al tools should aspire to have high healthcare value and avoid diverting resources from higher-priority areas
Al tools should consider the biographical drivers of health
Al tools should be designed to be easily tailored to the local population
Al tools should promote a learning healthcare system
Al tools should facilitate shared decision-making

ICMRA has also proposed general and specific recommendations for the EU (1) on how AI development and implementation monitoring activity should be exercised by specially created institutional bodies.

Specifically, the recommendations are synthetized in Tables 4, 5.

**Table 4:** ICMRA general recommendations.

**General Recommendations for AI**
Standing working group dealing with AI
Need for international guidelines (ICH)
Risk-based approach to assessing and regulating AI
Cooperation with existing ethics committees’ networks and AI expert groups
Collaboration among Medicines and Medical Devices Regulatory authorities

**Table 5:** ICMRA recommendations for EU.

**Recommendations for EU**
A clear legal definition of AI use in medicines development is needed
Establish clear mechanisms for regulatory cooperation between competent authorities and notified bodies for medicines and medical devices to facilitate the oversight of AI-based software intended for use with medicinal products
Device classification by the type of technologies used
Exchange information about clinical trials involving AI
Regulatory agencies as a trusted party and regulatory data custodian
In the post-marketing: notification or reassessment of data/cyber security and privacy measures
The post-authorization management may need to be adapted to accommodate updates to the AI software associated with a medicine
Consider which relevant features of the monitored data may influence the risk/benefit assessment by regulatory bodies

In June 2023, the European Parliament voted with a strong majority in favor of the Artificial Intelligence Act. The goal is to ensure compliance with the EU’s core values in the context of the use of AI, particularly the safety of users, respect for their privacy, and transparency and non-discrimination (21). It is clear from the AI Act that EU’s position is to consider medical devices implemented with AI as high-risk, as enunciated in Title III “High-Risk AI System,” a category encompassing all those technologies that may adversely affect fundamental human rights and, therefore, require stricter regulation by the relevant bodies.

The AI Act also provides in Title IV, Art. 52 “Transparency obligations for certain AI systems,” specifically the text states:

1. *“Providers shall ensure that AI systems intended to interact with natural persons are designed and developed in such a way that natural persons are informed that they are interacting with an AI system, unless this is obvious from the circumstances and the context of use. This obligation shall not apply to AI systems authorized by law to detect, prevent, investigate and prosecute criminal offenses, unless those systems are available for the public to report a criminal offense.”*
2. *“Users of an emotion recognition system or a biometric categorization system shall inform of the operation of the system the natural persons exposed thereto. This obligation shall not apply to AI systems used for biometric categorization, which are permitted by law to detect, prevent and investigate criminal offenses.”*
3. *“Users of an AI system that generates or manipulates image, audio or video content that appreciably resembles existing persons, objects, places or other entities or events and would falsely appear to a person to be authentic or truthful (‘deep fakes’), shall disclose that the content has been artificially generated or manipulated. However, the first subparagraph shall not apply where the use is authorized by law to detect, prevent, investigate and prosecute criminal offences or it is necessary for the exercise of the right to freedom of expression and the right to freedom of the arts and sciences guaranteed in the Charter of Fundamental Rights of the EU, and subject to appropriate safeguards for the rights and freedoms of third parties.”*

## Discussion

The system of care nowadays has to consider, based on the highlighted elements, two factors: (1) the therapeutic alliance between patient and caregiver, which sees its foundation in informed consent; (2) the intervention in the process of a third actor, the AI.

Informed consent represents the synthesis of two fundamental human rights, such as the right to health and the right to self-determination. Over the years, as a result of technological and scientific innovations, as well as cultural and social changes, the intrinsic nature of the relationship of care between doctor and patient has changed radically, transitioning from a paternalistic type of relationship, in which the doctor stood as the sole holder of decision-making power in the care path of his patient, to a true therapeutic alliance between doctor and patient, in which the latter’s decision-making autonomy takes on a fundamental role, thus becoming an active part in the care decision-making process. The main characteristics of informed consent to be effectively valid, include: the personalization of consent relative to the case in question; freedom on the part of the patient in accepting or rejecting the proposed treatment; completeness in the information provided, which must also be up-to-date; ease of understanding on the part of the patient of what is set forth, making the information comprehensible to the user; and the possibility of withdrawal of consent at any time during the course of care on the part of the patient.

Considering the above premises, for the advent of AI to actually materialize and integrate into the system of care, it must necessarily be accepted not only by the scientific community, but also by the individual patient, as a new tool potentially employed, therefore, its integration into the informed consent proposal is also essential.

Since informed consent is personal and therefore customized to the specific patient’s care pathway, it is necessary to highlight how the intervention of AI in the same pathway must also be considered in the individual steps proposed. However, this inevitably introduces several critical issues and questions, secondary to the inherent characteristics of AI, this being a rapidly advancing technology and above all whose mechanisms of operation are not in themselves characterizable at every single stage of its operation. In fact, one of the main problems in the use of AI in the context of informed consent is that of the “black box,” defined as the impossible transparency of the container, the AI. This makes its internal mechanisms, i.e., the learned patterns, not definable *a priori*, non-visible, which depending on the input provided and the phenomena experienced, can lead to different and unpredictable outcomes. This type of tool, characterized by an inherent non-intelligibility of operation together with the increasing level of autonomy, offers a different instrument from what has been used in health care to date, potentially increasing the standard of care, however, not completely predictable, thus laying the groundwork for an ethical and medico-legal dilemma between ensuring adherence to better standards and, on the other hand, the impossibility of completely controlling the machine, with possible repercussions on users (22).

It should also be pointed out that since the algorithms underlying AI are a product resulting from the activity of human beings, they may have acquired biases (defined as systematic errors in its outputs or processes (23, 24)), resulting from the socio-cultural heritage of the producer or from mere methodological errors during the design phase (25). While the use of AI can implement treatments and make them more accessible, it can also reinforce already existing disparities, perpetuating and reinforcing according to the learning model the biases inherent in the initial input provided by the source data (26). If a populational subgroup, for heterogeneous reasons, rejects the use of AI in its care pathway, the same technology not only could not be offered to these users, but would scotomize that group from the distribution analysis of the study variables. Therefore, this would lead to an exclusion/selection bias, subsequently potentially amplified by the autonomous learning mechanisms of the machine itself. So, dissent to the use of AI by patients could reverberate into a systematic selection bias with a distortion from the true representation of the epidemiological characteristics of the population under study, ultimately resulting in potential erroneous conclusions even in the field of medical research (27).

It seems appropriate to consider how AI can concretely integrate into the proposal for informed consent to treatment, responding to the dilemma between the patient’s self-determination and his right to health and the best available care.

It will be necessary to integrate AI in every step of the care pathway, from history collection to objective assessment, as well as in the clinical, laboratory, and instrumental diagnostic pathway, in therapeutic procedures (pharmacological, interventional, and/or surgical), and in the definition of prognosis and follow-up pathways.

After an exhaustive explanation of the possible uses of AI and how it works, at every possible node of the pathway of care and treatment the patient must have the option to choose whether to avail him of it or to renounce it, even at the expense of not having the opportunity to take advantage of the best standards of care. This possibility should be posed on a per-act basis, so as to maintain the personalization of consent and not have to accept or opt out tout court of the presence of the third-party actor. In these terms, it seems useful to propose guiding principles for optimal writing of informed consent in the new model of care toward which we are inevitably moving.

Key proposals for informed consent in the era of triangular therapeutic alliance between physician, patient, and artificial intelligence:

1. The patient, consistent with the nature of “black boxes,” needs to understand what AI is and how it works.
2. The possibility of withdrawal of consent at any time and optimal privacy management must be guaranteed; the data used must not be traceable to the patient unless explicitly requested by the patient.
3. It must be defined in which nodes AI intervention is proposed, and the patient must be able to choose in which of these to accept or reject it.
4. The role of AI in each individual node must be identified, breaking it down into types of activities performed and level of autonomy in managing them.
5. The consequences of accepting or rejecting AI in each individual treatment step must be made explicit.
6. During each medical act, the patient should be accompanied, explaining to him which activities are performed by the AI and which by the Physician, as well as their respective roles.
7. Adequately trained individuals should be provided to cooperate in drafting and administering consent, technical-procedural explanation, as well as lending assistance in case of ethical dilemmas.

As shown in Table 6, those are some examples for each of the above key points:

**Table 6:** Examples for key points.

**Key points**	**Examples**
Nature of AI and black box	In the case of diagnostic hypotheses, the patient needs to understand how they are generated by AI models and not by the health care provider, not necessarily having to understand every single technical step that led to the proposed outcome.
Withdrawal of consent and privacy	During a follow-up, operated by AI, the patient must be able to choose at any time to continue the same course by interfacing only with the physician, thereby terminating AI’s participation, as well as revoking AI’s access to his or her data.
When AI intervenes in the care pathway	The patient may choose to undergo an AI-operated procedural intervention, such as the placement of valve prostheses, however, refuse to have AI itself involved in the subsequent follow-up
AI activities and autonomy in individual steps	When reading radiological images, it must be clear, for example, whether the report is written by the physician or by the AI itself, as well as whether the image is interpreted by the AI or the AI only served an alert function for suspicious patterns.
Consequences of accepting or rejecting AI	Explain to the patient how access to AI can, in some cases, ensure a better procedural outcome by hesitating a better risk/benefit ratio.
Illustration of physician/AI roles	Delineate, as far as possible, a clear boundary between what are the duties of the physician and AI, specifying who performs the act and who supervises it.
Multidisciplinary teams	If a patient is undecided about the possible use of AI in his or her course of treatment, he should have access to information of a technical nature, as well as discussion with support in ethics.

Artificial intelligence is a current reality, destined to become an integral part of the treatment process for doctor and patient, therefore, since it cannot be scotomized, the challenging goal will be to explain its nature and applications to the patient to ensure consent/dissent, which cannot be inherently informed regarding the inner workings of AI but rather must consider its impact in the possibility of treatment. The goal, then, to be pursued progressively and collectively with the inevitable technological development, guaranteeing both the right to Health and Self-determination, is the building of a therapeutic alliance between physician, patient and AI.

Finally, two questions arise spontaneously, only partially provocative:

- Will some physicians refuse the use of AI for ethical reasons?
- Will the physician ever risk being excluded from this new triangular therapeutic alliance?

## Author contributions

RS: Conceptualization, Writing – original draft. RT: Conceptualization, Writing – original draft. FA: Writing – review & editing. ST: Writing – review & editing. DD: Writing – review & editing.
